# A systematic review on influence of printing layer thickness on the marginal and internal fit of 3D-printed fixed dental prostheses

**DOI:** 10.1007/s10266-025-01241-y

**Published:** 2025-11-20

**Authors:** Walid Abdelhady, Dina Abozaid, Maged Mohammed, Mohammed Ashraf, Mohamed Metwally, Hussein Mohammed

**Affiliations:** 1 Crown and Bridge Department, Faculty of Dental Medicine, Al Azhar University, Cairo, Egypt; 2 Dental Biomaterials Department, Faculty of Dentistry, Tanta University, Tanta, Egypt; 3 Faculty of Dental Medicine, Al Azhar University, Cairo, Egypt; 4 Medical Research Group of Egypt, Negida Academy LLC, Massachusetts Avenue, Arlington, MA 02474 USA

**Keywords:** 3D printing, Additive manufacturing, Fixed dental prosthesis, Marginal fit, Layer thickness, CAD/CAM

## Abstract

The purpose of this study is to systematically review the effect of printing layer thickness and build orientation on the marginal and internal fit of 3D-printed fixed dental prostheses (FDPs). A systematic search was conducted in PubMed, Scopus, Web of Science, and Cochrane Library up to August 2025 following PRISMA guidelines (PROSPERO: CRD42024591005). Comparative studies assessing the impact of layer thickness on marginal gap (MG), internal gap (IG), or absolute marginal discrepancy (AMD) of 3D-printed FDPs and crowns were included. Risk of bias was assessed using a modified CONSORT tool. Thirteen in vitro studies met the eligibility criteria. Thinner layers (20–50 μm), particularly at 50 μm, improved marginal and internal fit compared to thicker layers (100 μm). Intermediate build orientations (45°–60°) minimized discrepancies, while extreme orientations (0° and 90°) increased stair-stepping effects. Marginal gap values generally ranged from 40 to 120 μm, within clinical acceptability. Subtractive (milled) restorations consistently showed superior fit compared to additive manufacturing. Layer thickness and build orientation significantly influence the fit of 3D-printed FDPs. A layer thickness of 50 μm at intermediate build orientations (45°–60°) provides the best adaptation. Milling remains the gold standard for precision, although optimized 3D-printing parameters can achieve clinically acceptable accuracy. Optimizing 3D-printing parameters—especially using 50 μm layer thickness with intermediate build orientations—enhances the marginal and internal precision of FDPs. While 3D printing is suitable for provisional restorations, milled prostheses remain preferable for definitive restorations requiring the highest accuracy.

## Background

Dental prosthesis manufacture has been transformed by digital impressions and computer-aided design/computer-aided manufacturing (CAD/CAM) systems, which have supplanted conventional procedures such as the lost wax process [1]. The lost wax method is expensive and time-consuming, requiring a prosthesis to be shaped in wax and then cast following investment, with prosthesis quality heavily dependent on the technician’s skill [2, 3]. In contrast, CAD/CAM techniques offer a lower learning curve, rapid production of high-quality prostheses, and consistent results, making them widely applicable in dentistry [4–6].

CAM methods fall into two categories: additive manufacturing (AM) and subtractive manufacturing (SM) [7]. In SM, a block or blank of material is milled into the required shape, a process that generates significant waste, wears down milling tools, and may induce micro-cracks in restorations [8, 9]. Conversely, AM, commonly referred to as 3D printing, constructs restoration layer by layer, overcoming many of the limitations associated with SM [10, 11]. Digital light processing (DLP) and stereolithography (SLA) are two AM technologies that are especially common in dentistry. SLA employs a laser to polymerize photosensitive resins, achieving high accuracy but with higher costs and longer production times compared to DLP [12, 13].

Several parameters influence the accuracy of AM prostheses, including layer thickness, build orientation, post-processing protocols, light exposure duration, and x–y resolution [14–17]. Among these, layer thickness and build orientation have been identified as critical factors affecting the surface roughness, geometric accuracy, and marginal precision. Studies have demonstrated that optimizing these parameters significantly enhances the adaptation and durability of prostheses [18, 19].

Accurate marginal precision is paramount to sustained success of fixed dental prosthesis. Marginal misfit can lead to cement dissolution, bacterial infiltration, secondary caries, and periodontal complications, jeopardizing the prosthesis and surrounding tissues [20, 21]. While the American Dental Association (ADA) advises type I luting cements to have marginal gaps of ≤ 25 µm and type II cements to have marginal gaps of ≤ 40 µm. [22], Marginal gaps under 120 µm are clinically acceptable, according to McLean and von Fraunhofer's suggestion [23].

Provisional restorations are essential during the transition to final restorations, providing soft-tissue management, pulp protection, positional stability, and aesthetics. Polymethylmethacrylate (PMMA) is a commonly used material for these temporary restorations due to its versatility. However, PMMA restorations fabricated directly in the oral cavity are susceptible to polymerization shrinkage and thermal trauma, which can lead to dimensional inaccuracies [24]. Indirect fabrication methods offer several advantages, including improved marginal precision, enhanced mechanical properties, better color stability, and reduced technique sensitivity [21, 25].

Despite these advancements, a comprehensive analysis of how layer thickness affects the precision and clinical viability of additive manufactured dental prosthesis remains limited. This systematic review aimed to evaluate the effects of layer thickness on the marginal and internal fit of additively manufactured fixed dental prosthesis (AM-FDPs).

## Methods

This systematic review was conducted in accordance with the Preferred Reporting Items for Systematic Reviews and Meta-Analyses (PRISMA) guidelines [26]. The protocol was prospectively registered in the PROSPERO database (Registration No. CRD42024591005).

### Eligibility criteria and search strategy

Using PICO format, the criteria included patients requiring fixed dental prostheses (FDPs) as the population (P), variations in layer thickness in additive manufacturing as the intervention (I), standard layer thicknesses as the comparator (C), affect the marginal and internal fit of the dental prostheses as the outcome (O).

Studies were included if it:

- compared in vitro or in vivo studies on fixed dental restorations (crowns or FDPs)
- investigated the effect of different 3D-printing layer thicknesses
- reported quantitative data on at least one of the following:
  o Marginal Gap (MG): Shortest perpendicular distance from the restoration’s internal surface to the abutment margin
  o Internal Gap (IG): Mean distance across all internal measuring points
  o Absolute Marginal Discrepancy (AMD): Linear distance from the most peripheral end of the restoration margin to the abutment finish line
- published in English, with full-text availability.

Studies were excluded if:

- unrelated to the PICO question
- research on removable prostheses, systematic reviews, case reports, or animal studies
- outcomes other than MG, IG, or AMD
- full text unavailable or not in English.

A comprehensive electronic search was conducted in August 2025 across four major databases: PubMed, Scopus, Web of Science, and the Cochrane Central Register of Controlled Trials. The search strategy combined keywords and Boolean operators related to printing parameters, marginal and internal fit, and additive manufacturing. The complete search strings for each database are provided in Table 1.

**Table 1:** The search strategy in the different databases for the study

Search strategy	Databases
PubMed Web of Science Scopus Cochrane library	((“printing parameter” OR “layer thickness” OR “layer depth” OR “layer size” OR “layer parameter” OR thickness) AND (“marginal adaptation” OR “marginal fit” OR “passive fit” OR accuracy OR trueness OR precision OR retention OR “prosthesis retention” OR “dimensional measurement accuracies” OR “dimensional measurement accuracy” OR “trueness analysis” OR “internal discrepancies” OR “internal discrepancy” OR “marginal discrepancies” OR “marginal discrepancy” OR “internal fit” OR “marginal fit” OR “marginal gap”) AND (“additive manufacturing” OR “rapid prototyping” OR “3D printing” OR “3-dimensional printing” OR “three dimension printing” OR “selective laser melting” OR print OR “RP techniques” OR manufacturing OR stereolithography OR “digital light processing technology”) AND (“implant-supported framework” OR “implant retained fixed partial dentures” OR “single implant crowns” OR “complete arch implant supported prostheses” OR "dental crown" OR "fixed partial denture" OR "dental crown" OR crown OR "dental bridge" OR bridge OR "fixed restoration" OR “dental prosthesis” OR “tooth crown” OR "dental restoration” OR “dental restoration, temporary” OR “provisional fixed partial denture” OR “provisional crown” OR “provisional dental restoration" OR “provisional bridge” OR “temporary dental restoration” OR " interim dental crown" OR " interim fixed partial denture"))

A summary of the search strategy is presented in Table 1.

### Study selection

All retrieved records were imported into Rayyan—Intelligent Systematic Review (Rayyan QCRI, Free Web Version; Qatar Computing Research Institute, Doha, Qatar) [27] to facilitate blinded screening by multiple reviewers. Title and abstract screening was followed by full-text evaluation, applying the predefined eligibility criteria (Table 1). Discrepancies were resolved through discussion, with a third reviewer consulted when necessary.

### Data extraction

Two reviewers (M.M and MA) independently extracted data using a pre-designed form. Extracted variables included:

- study characteristics (design, country)
- restoration material and type (interim or definitive)
- fabrication techniques, printer model, layer thickness, and build orientation
- post-curing protocol and thermocycling conditions
- measurement methods (e.g., microscopy, micro-CT, digital superimposition)
- quantitative results for MG, IG, and/or AMD.

If data were missing and could not be retrieved from the authors, those variables were excluded from the quantitative synthesis without imputation.

### Quality assessment method

The methodological quality and risk of bias of the included studies were independently assessed using the Modified CONSORT Scale [28, 29]. This tool comprises 14 items, each scored as “Yes” (criterion met) or “No” (criterion not met), with a maximum score of 14. Risk of bias was categorized as:

- low risk: 11–14 points
- moderate risk: 7–10 points
- high risk: 0–6 points.

Any disagreements were resolved through discussion and consensus, with a third reviewer (W.A) consulted when necessary.

## Results

### Study characteristics

**Fig. 1:**
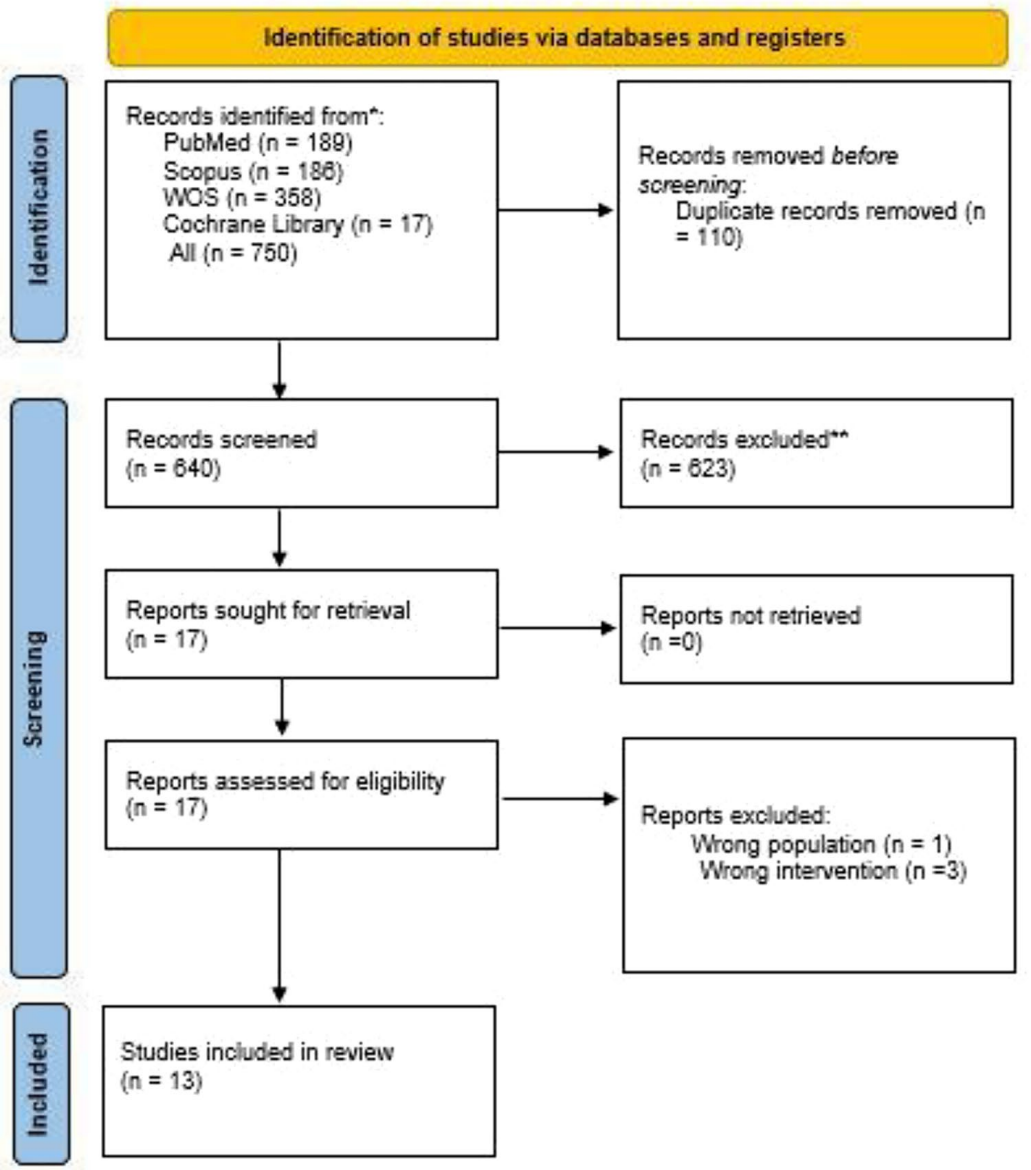
PRISMA flowchart

The systematic search initially yielded 750 studies. After removing duplicates, 640 studies remained. Title and abstract screening led to the exclusion of 623 articles. The remaining 17 articles were assessed in full text based on the predefined inclusion and exclusion criteria, resulting in the exclusion of 4 studies as presented in the PRISMA flowchart (Fig. 1). Ultimately, 13 studies met the eligibility criteria and were included in this review. The extracted data and qualitative comparisons are presented in Tables 2, 3. Due to substantial heterogeneity in research methodologies and study designs, a meta-analysis was not conducted. Excluded studies and there causes showed in supplemental materials (Table 1S).

**Table 2:** Summary of extracted data from the included studies

References	Aim of the study	Restoration type	Material of the restoration (brand)	Model prepared	Scanner	3D printer	Examination method for precision	Accuracy measurement	Cement space (µm)	Conclusion
Jang, Kim et al. 2024 [30]	To evaluate the fit according to the build orientations and layer thicknesses in SLA manufactured 3-unit resin prostheses	3-unit FPD	PMMA (ZMD-1000B; Dentis)	PMMA Model (Yamahachi dental Mfg)	Laboratory scanner (Medit T500; Medit Corp)	SLA 3D printer (ZENITH U; Dentis)	Marginal fit (AMD, MG And Internal fit)	µCT (SkyScan 1172; Bruker)	100µ	The best settings for fit (layer thickness: 50 µm, angles: 45° or 60°). Both 3D-printed (SLA) and milled prostheses fit well enough for clinical use
Daou 2022 [31]	To evaluate the marginal and internal fit of Co-Cr 3-unit frameworks fabricated by additive manufacturing with different melting layer thicknesses on different abutments	3-unit FPD	Co-Cr metal powder (Mediloy S-Co)	Typodont Model (Frasaco A3; Frasaco)	Laboratory scanner (Ceramill map 400; Amann Girrbach AG)	Laser machine (SLM 100; ReaLizer GmbH)	Marginal Fit Internal Fit	Silicone replica technique. (SZX-ZB7; Olympus) At 20 X	30µ	Additive manufacturing produced metal frameworks with acceptable fit; discrepancies correlated with lamination layer thickness
Çakmak, Cuellar et al. 2024 [32]	Compared the effect of printing layer thickness on the trueness of 3-unit interim FPDs fabricated using additive manufacturing with that of those fabricated by subtractive manufacturing	3-unit FPD	PMMA (NextDent C&B MFH; 3D Systems)	Typodont Model (ANA-4; frasaco GmbH)	Intraoral scanner (Medit i500 v. 1.2.1, Medit, Seoul, Korea)	DLP printer (MoonRay S100; SprintRay Inc)	Truness (RMS)	Superimposition of 2 different datasets	30µ	Printing layer thickness influenced the trueness of 3D-printed interim FDPs. 20 µm and 50 µm layer thicknesses resulted in higher trueness compared to 100 µm. Milled interim FDPs showed superior trueness over 3D-printed restorations, irrespective of layer thickness
Hasanzade, Yaghoobi et al. 2023 [33]	Compared the effect of printing layer thickness on the marginal and internal fit of interim crowns	Crowns	UDMA (PowerDent Temp resin, Protech)	Typodont tooth (Nissin Dental Products)	Laboratory digital scanner (Shining 3D ex pro)	DLP‐based 3D printer (Digident Plus)	marginal fit internal fit	Silicone replica technique. Replica (Olympus BX60; Olympus Optical) At 12.5X	80µ	3D-printing layer thickness significantly impacts marginal and internal fit A 50 µm layer thickness resulted in the best overall adaptation. For occlusal areas, a 25 µm layer thickness was the most suitable
Yang, Kim et al. 2022 [34]	To analyze the marginal fit of three-unit resin prostheses printed with the stereolithography (SLA) method in two build orientations (45°, 60°) and two-layer thicknesses (50 μm, 100 μm)	3-unit FPD	UDMA (ZMD-1000B temporary; Dentis, Daegu, Korea)	PMMA Model (Yamahachi Dental MFG, Ochigara, Japan)	Model scanner (T500; Medit, Seoul, Korea)	SLA 3D printer (Zenith U; Dentis, Daegu, Korea)	Marginal fit (AMD and MG)	µCT (Skyscan 1172; Bruker Micro-CT, Billerica, MA, USA)	100µ	Build orientation significantly influenced marginal fit, with 45° showing the best results. Three-unit resin prostheses fabricated using SLA exhibited poor marginal adaptation near the pontic. No significant differences were found between different layer thicknesses, necessitating further research
Çakmak, Cuellar et al. 2021 [35]	Investigated the trueness and margin quality of interim crowns printed with three different layer thicknesses (20, 50, and 100 μm) compared to milled PMMA crowns	Crowns	PMMA (Nextdent Crown and Bridge Micro Filled Hybrid-MFH, C&B; 3D systems, Soesterberg, The Netherlands)	Typodont Model (ANA-4, Frasaco GmbH, Tettnang, Germany)	Intraoral scanner (Medit i500 v. 1.2.1, Medit, Seoul, Korea)	DLP printer (MoonRay S100, SprintRay Inc, Los Angeles, CA, USA)	Truness (RMS)	Superimposition of 2 different datasets	30µ	Printing layer thickness affected the trueness and marginal quality of 3D-printed interim crowns. A thickness of 20 or 50 µm provided better trueness and margin quality than 100 µm. Material and printing settings played a role in determining the optimal thickness
(Park, Kim et al. 2019 [36]	Investigated the influence of 3D-printing parameters on the fit and internal gap of 3D-printed resin dental prostheses	3-unit FPD (Two-implant abutment)	PMMA (NextDent C&B (3D systems, Soesterberg, The Netherlands)	PMMA Model (Yamahachi dental Mfg)	Model scanner (Freedom HD; DOF, Seoul, Korea)	DLP printer (D2-120, Hephzibah, Incheon, Korea)	Marginal Fit (AMD and MG) Internal Fit	µCT (Skyscan 1172; Bruker micro-CT, Kontich, Belgium) at 100 × magnification	24µ	Build orientation and layer thickness affected the fit of 3D-printed prostheses Orientations of 45° and 60° provided optimal marginal fit and internal gap adaptation. The marginal fit of 100 µm and 50 µm layer thicknesses was comparable With optimized parameters, 3D-printed prostheses achieved a fit comparable to milled restorations. Both 3D-printed and milled prostheses were within clinically acceptable marginal fit limits
Kaleli, Ural et al. 2019 [37]	Compared the marginal and internal adaptation of laser-sintered cobalt-chromium single crown frameworks fabricated with layer thicknesses of 25 µm and 50 µm	Crowns (Metal copy)	Co-Cr dental alloy powder (Starbond CoS Powder 30; S&S Scheftner GmbH)	Resin Models (E-Model; EnvisionTEC GmbH)	Desktop scanner (AutoScan-DS200 + ; Shining 3D Tech)	DLP printer (VIDA; EnvisionTEC GmbH)	Marginal fit (MG)	Silicon replica Examined with a stereomicroscope (SZX16; Olympus) at × 40 magnification	30µ	Sintering layer thickness did not significantly affect metal framework adaptation Mean marginal discrepancy was below 120 µm in all groups Mean internal discrepancy was below 50 µm in direct metal laser melting groups Since no significant difference was observed between sintering parameters, a 50 µm layer thickness is recommended for fabricating thin margins of laser-sintered restorations
Morón‐Conejo, Berrendero et al. 2024[38]	To investigate the fit of provisional crowns fabricated using DLP-based 3D printing in an open and proprietary manufacturing workflow, in comparison to milling	interim crown	PMMA Block Huge A3, Shandong Huge Dental Material Corporation, Chandong, China) for milled group, Sprintray EU Temporary Crown & Teeth A3 resin (Pro3dure medical GmBH, Iserlohn, Germany) for the SR100 group, and VarseoSmile Temp A3 resin (BEGO Bremer Goldschlägerei Wilh. Herbst GmbH & Co., Bremen, Germany) for the other printed group	A maxillary typodont (AG-3; Frasaco GmbH, Tettnang, Germany)	PrimeScan intraoral scanner (Dentsply Sirona, USA)	SprintRay (Pro95, CA, USA)	1-marginal fit 2- internal fit	Silicone replica technique Replica by a stereomicroscope (M-125, Leica, Bensheim, Germany) at magnification factor 4	50	All groups of interim crowns fabricated with 3D printing and milling technology demonstrated a clinically acceptable fit, with the exception of the SR100 group. The thickness of the layer appears to be a crucial factor, with thinner layers resulting in superior fit outcomes, comparable to those observed in the gold standard milled crowns
(Ghorbanpour Arani, Hasanzade et al. 2025[39]	Compare the marginal and internal adaptation of polymethyl methacrylate interim fixed partial denture (FPD) restorations fabricated by three-dimensional (3D) printing with different layers thicknesses	3-unit FPD	Freeprint Temp resin (Detax GmbH & Co. KG, Ettlingen, Germany)	Nickel–chromium metal dies (Damcast NP; Damcast Dentalloy Corporation, Zhengzhou, China) were fabricated by a milling machine (MaP-400; Ceramill; Amann Girrbach AG, Koblach, Austria)	Shining 3D EX Pro scanner; Shining 3D Tech. Co., Ltd., Hangzhou, China)	a DLP printer (Asiga UV Max; Asiga, Sydney, Australia)	1-marginal fit 2- internal fit	Silicone replica technique. replica examined by a stereomicroscope (Leica EZ4D; Leica Microsystems GmbH, Wetzlar, Germany) at 5.12 × magnification	50	Different printing layer thicknesses affected the marginal and internal gap. According to the present results, the 50 µm layer thickness resulted in the highest adaptation of interim 3-unit FPD restorations
Dönmez, Demirel et al. 2025[40]	Evaluate the fabrication and fit accuracy of AM three-unit FPDs, using a definitive resin, compared to a subtractively manufactured FPD in high-impact polymer composite	Three-unit fixed partial dentures	Interim resin (VarseoSmile Triniq; Bego, Bremen, Germany)	maxillary typodont (Dentsply Sirona, Bensheim, Germany)	industrial scanner (Artec Micro; Artec 3D, Luxembourg City, Luxembourg)	(Max UV; Asiga, Sydney, Australia)	root mean square (RMS) method	triple scan protocol, which aligns the T-FPD-STLs, FPD-M-STLs, and M-STL in the same coordinate system after consecutive superimpositions	100	FPDs additively manufactured with 30- and 90-degree build orientation and 50-micron layer thickness had accuracy similar to SM-CR FPDs. However, SM-CR FPDs had better fit than AM FPDs
Jang, Kim et al. 2020[41]	To investigate the optimal cement space in the 3D-printed 3-unit resin fixed partial denture	Two-implant-supported 3-unit provisional FPD	Temporary resin (Zenith ZMD-1000B; Dentis, Daegu, Korea)	Polymethylmethacrylate (PMMA) resin block (Yamahachi dental MFG; Ochigara, Japan) was milled with 5-axis milling machine (IDC MILL 5X; Amann girrbach AG, Koblach, Austria) using the STL file	The model was designed in software and milled	SLA3Dprinter(ZenithU;Dentis,Daegu,Korea)	1-marginal fit 2- internal fit	Micro-CT scanner (Skyscan 1172; Bruker micro-CT, Kontich, Belgium)	90	For the SLA 3D-printed 3-unit resin fixed partial denture, setting the cement space to 90 µm or less is not recommended due to the interference in seating
Mou, Zhong et al. 2024[42]	Investigated the effects of build angle and layer thickness on the trueness and precision of zirconia crowns manufactured using digital light processing (DLP) technology	Single crowns	3Y-TZP (ADT-ZrO2-THP01-A; Shenzhen Adventure Tech Co. Ltd., China)	Standardized resin dental model of a mandibular first molar	Temporary resin (Zenith ZMD-1000B; Dentis, Daegu, Korea)	DLP printer (ADT-3D-ZP-Printer-Pro-192–50, Shenzhen Adventure Tech Co. Ltd., China)	Root mean square (RMS)	Superimposition with the reference scan data	70	The results demonstrated that the build angle and layer thickness significantly affect the accuracy of zirconia crowns printed by DLP technology. A layer thickness of 30 μm provided superior accuracy compared to that of 50 μm. To ensure margin accuracy, a layer thickness of 30 μm and build angle of 0◦ or 45◦ are recommended for DLP printing of zirconia crowns

**Table 3:** Marginal and internal fit measurements (μm) reported in the included studies

References	Groups	Marginal precision			Internal fit					
			Mean ± SD	Mean ± SD	Mean ± SD		Mean ± SD		Mean ± SD	
[30]		AMD	MG	CE	AX		LA		OC	
	Group 1a: 50 μm 0°	81.11 ± 22.58	42.91 ± 13.13	120.99 ± 16.96	114.78 ± 16.77		68.73 ± 7.37		113.87 ± 34.88	
	Group 1b: 50 μm 30°	74.17 ± 13.98	44.73 ± 14.07	99.36 ± 8.33	101.069 ± 11.72		74.44 ± 21.84		97.25 ± 31.98	
	Group 1c: 50 μm 45°	71.81 ± 13.62	40.36 ± 7.51	85.38 ± 10.48	89.13 ± 12.64		77.196 ± 10.101		125.70 ± 22.53	
	Group 1d: 50 μm 60°	105 ± 20.43	62.18 ± 17.2	66.97 ± 11.72	78.002 ± 10.57		75.82 ± 9.56		147.96 ± 25.44	
	Group 1e: 50 μm 90°	120.69 ± 16.84	83.39 ± 14.39	84.3 ± 14.49	84.41 ± 8.27		66.296 ± 10.37		173.03 ± 23.26	
	Group 2a: 100 μm 0°	94.58 ± 22.21	60.12 ± 22.83	113.23 ± 9.25	108.19 ± 12.87		83.23 ± 10.37		205.42 ± 21.08	
	Group 2b: 100 μm 30°	95.28 ± 22.22	61.7 ± 15.01	104.5 ± 11.41	112.56 ± 5.74		101.22 ± 8.46		171.06 ± 13.81	
	Group 2c: 100 μm 45°	78.61 ± 10.39	52.36 ± 11.88	90.76 ± 14.18	102.49 ± 18.38		100.37 ± 13.11		156.69 ± 42.15	
	Group 2d: 100 μm 60°	75.69 ± 10.39	52.61 ± 12.2	82.51 ± 14.49	91.63 ± 4.595		83.44 ± 14.47		133.87 ± 15.26	
	Group 2e: 100 μm 90°	116.25 ± 15.77	80.73 ± 17.2	100.08 ± 12.95	112.02 ± 12.18		65.03 ± 7.37		150.77 ± 26.89	
[31]		Overall marginal fit		Axial			occlusal			
	Group 1: 25 μm	49.89 ± 12.32		66.2 ± 12.45			137.50 ± 11.84			
	Group 2: 50 μm	63.79 ± 10.74		70 ± 10.78			140.54 ± 10.62			
	Group 3: 100 μm	68.84 ± 10.74		87 ± 11.23			154.50 ± 11.69			
[32]		Overall marginal fit		Intaglio RMS			Intaglio Occlusal (RMS(			
	Milling group	4 ± 2.26		18.8 ± 7.89			2.9 ± 0.74			
	Group 1: 20 μm, 45°	4.7 ± 1.42		17 ± 3.13			30.7 ± 5.31			
	Group 2: 50 μm, 45°	3.1 ± 1.1		15.1 ± 4.9			31.1 ± 4.2			
	Group 3: 100 μm, 45°	3.1 ± 1.1		22 ± 6.77			38.2 ± 4.54			
[33]		Overall marginal fit		Axial			axio-occlosal		occlusal	
	Group 1: 25 μm, 45°	206.5 ± 66.22		174.29 ± 30.7			222.89 ± 30.73		282.83 ± 53.73	
	Group 2: 50 μm, 45°	159.93 ± 11.74		147.62 ± 17.57			174.33 ± 21.75		420.45 ± 92.41	
	Group 3: 100 μm, 45°	232.39 ± 14.07		163.39 ± 12.51			225.54 ± 14.35		697.2 ± 82.92	
[34]		AMD	MG							
	Group 1: 50 μm 45°	81.52 ± 4.94	74.71 ± 2.91							
	Group 2: 50 μm 60°	87.78 ± 4.94	82.93 ± 2.22							
	Group 3: 100 μm 45°	90.097 ± 4.94	86.23 ± 3.82							
	Group 4: 100 μm 60°	82.74 ± 4.29	77.34 ± 2.85							
[35]		Overall marginal fit		Intaglio RMS			Intaglio Occlusal (RMS)			
	Milling group	11.3 ± 14.36		32.6 ± 15.01			31.5 ± 6.92			
	Group 1: 20 μm	14.9 ± 9.57		49.9 ± 12,13			33.4 ± 2.22			
	Group 2: 50 μm	9.1 ± 8.02		45.4 ± 15.75			34.7 ± 1.83			
	Group 3: 100 μm	14.6 ± 9.94		52.8 ± 17.32			41.5 ± 12.55			
[36]		AMD	MG	Cv	AX		AN		OC	
	Group 1a: 50 μm 0°	236.96 ± 22.82	46.54 ± 28.25	328.48 ± 28.15	252.04 ± 33.36		121.21 ± 22.68		102.06 ± 12.73	
	Group 1b: 50 μm 30°	211.33 ± 18.14	43.39 ± 27.9	296.97 ± 20.33	240.87 ± 11.63		131.47 ± 26.08		148.13 ± 26.54	
	Group 1c: 50 μm 45°	185.71 ± 19.89	58.73 ± 21.19	283.64 ± 28.15	203.56 ± 18.19		139.23 ± 25.33		171.22 ± 35.47	
	Group 1d: 50 μm 60°	169.84 ± 13.46	64.2 ± 18.37	260 ± 22.67	192.87 ± 11.63		139.96 ± 29.86		185.81 ± 15.43	
	Group 1e: 50 μm 90°	250.57 ± 26.33	137.99 ± 18.37	263.03 ± 21.11	197.58 ± 39.43		164.14 ± 41.204		207.12 ± 36.83	
	Group 2a: 100 μm 0°	200.23 ± 17.55	53.94 ± 6.71	312.12 ± 27.36	287.31 ± 23.25		122.27 ± 16.35		110.77 ± 16.79	
	Group 2b: 100 μm 30°	161.45 ± 29.84	52.57 ± 22.603	302.42 ± 10.16	252.63 ± 21.23		146.84 ± 44.41		166.18 ± 30.87	
	Group 2c: 100 μm 45°	161.905 ± 38.03	53.11 ± 27.901	295.45 ± 19.55	250.48 ± 21.74		151.92 ± 26.24		169.54 ± 26.81	
	Group 2d: 100 μm 60°	134.92 ± 15.21	47.09 ± 19.78	258.48 ± 26.58	209.92 ± 25.27		152.31 ± 26.44		192.11 ± 14.89	
	Group 2e: 100 μm 90°	242.86 ± 19.89	73.24 ± 31.43	227.58 ± 21.11	200.71 ± 15.16		141.36 ± 19.78		193.16 ± 31.14	
[37]		Overall marginal fit		Internal discrepancy						
	Lost wax group	77 ± 10		73 ± 9						
	Group 1: 25 μm	59 ± 6		46 ± 4						
	Group 2: 50 μm	58 ± 8		43 ± 2						
[38]		Global Fit (μm)		Marginal fit						
	Group mill	125.47 ± 74.86		70.77 ± 46.03						
	Group SR100:100 μm	217.53 ± 115.09		209.78 ± 97.84						
	Group B100: 100 μm	144.8 ± 74.61		122.25 ± 54.55						
	Group B50: 50 μm	123.87 ± 67.42		117.65 ± 70.42						
[39]		Premolar	Molar	Occlusal		Axial		Cervical		
				Premolar	Molar	Premolar	Molar	Premolar		Molar
	Group: 25 μm	172.91 ± 62.21	29.32 ± 110.44	345.62 ± 232.39	506.91 ± 145.7	115.81 ± 32.23	159.57 ± 32.34	194.58 ± 59.34		347.03 ± 90.43
	Group: 50 μm	126.35 ± 47.70	294.07 ± 104.76	241.41 ± 53.68	457.97 ± 114.2	92.12 ± 20.32	147.54 ± 33.56	164.96 ± 41.97		321.80 ± 86.69
	Group: 100 μm	228.53 ± 79.40	388.62 ± 84.35	357.07 ± 97.55	615.87 ± 122.10	112.42 ± 34.35	169.23 ± 35.90	259.68 ± 67.27		421.25 ± 72
[40]				Intaglio surface deviations						
	50 μm 0 degree			35.6 ± 2.1						
	50 μm 30 degree			32.1 ± 2.3						
	50 μm 45 degree			34.2 ± 4.8						
	50 μm 90 degree			31.4 ± 2.6						
	100 μm 0 degree			48.8 ± 3.8						
	100μm30 degree			50 ± 1.8						
	100μm45 degree			36.4 ± 3						
	100 μm 90 degree			40.9 ± 2.8						
	milling group			21.6 ± 1.2						
[41]				Axial gap		Occlusal gap				
				AX		AN		OC		
	CS 90 µm: 50 μm 0 degree	74.3 ± 36.9		116.5 ± 62.2		94.2 ± 42.3		174 ± 23		
	CS 90 µm: 50 μm 30 degree	65.7 ± 29.5		99.8 ± 55.1		89 ± 47.8		158.8 ± 29.2		
	CS 90 µm: 50 μm 45 degree	80.2 ± 34.3		69.2 ± 40.3		82.1 ± 41.5		153.6 ± 32.6		
	CS 90 µm: 50 μm 60 degree	80.2 ± 34.3		75.2 ± 45.1		59.1 ± 32.6		135.5 ± 23.5		
	CS 90 µm: 50 μm 90 degree	89.7 ± 40.9		62.9 ± 50		35.8 ± 35		111.1 ± 32.3		
	CS 90 µm: 100 μm 0 degree	126.4 ± 53.3		114.3 ± 72.4		105.5 ± 81		257.8 ± 43.2		
	CS 90 µm: 100 μm 30 degree	111.9 ± 32.6		101 ± 51.7		100 ± 70.9		196.5 ± 33.9		
	CS 90 µm: 100 μm 45 degree	87.4 ± 37.6		92.1 ± 58.1		96.7 ± 64.6		193.3 ± 25.7		
	CS 90 µm: 100 μm 60 degree	86.5 ± 44.6		102.5 ± 59.1		83.9 ± 43.7		163.8 ± 45		
	CS 90 µm: 100 μm 90 degree	95 ± 23		94 ± 57.5		58.7 ± 47.1		123.2 ± 39.5		
	CS 100 µm: 50 μm 0 degree	57.1 ± 33.2		126.1 ± 62.8		91 ± 52		166.2 ± 22		
	CS 100 µm: 50 μm 30 degree	63.3 ± 41.5		123.2 ± 56.9		100.7 ± 52.4		179.2 ± 25.7		
	CS 100 µm: 50 μm 45 degree	58.6 ± 40.8		89.5 ± 55.6		103.5 ± 49		153.7 ± 30.9		
	CS 100 µm: 50 μm 60 degree	57.8 ± 21		89.7 ± 52.5		90.8 ± 38.9		164.9 ± 31.1		
	CS 100 µm: 50 μm 90 degree	63.1 ± 24.5		98.9 ± 59.6		54.2 ± 36.5		106.7 ± 27.5		
	CS 100 µm: 100 μm 0 degree	79.4 ± 53.1		135.7 ± 68.2		101 ± 70.1		220.7 ± 50		
	CS 100 µm: 100 μm 30 degree	79.4 ± 38.1		127 ± 53.8		120 ± 54.5		192.8 ± 37.1		
	CS 100 µm:100 μm 45 degree	79.5 ± 53.2		116 ± 63.4		118.3 ± 67.1		176.8 ± 33.4		
	CS 100 µm: 100 μm 60 degree	77.3 ± 38.1		94.6 ± 66.1		102.8 ± 43.3		172.2 ± 39.8		
	CS 100 µm: 100 μm 90 degree	75.7 ± 34.2		139.6 ± 71.7		47.5 ± 28.1		84.7 ± 29.1		
[42]		Marginal trueness (RMS)		Intaglio trueness (RMS)						
	30 μm 0 degree	32.2 ± 3.2		43.8 ± 4.3						
	30 μm 45 degree	33.9 ± 2.4		48.1 ± 3.7						
	30 μm 90 degree	44.2 ± 3.2		37.4 ± 4						
	50 μm 0 degree	44.9 ± 2.5		70.7 ± 4.3						
	50 μm 45 degree	41.6 ± 4.1		61.7 ± 3.8						
	50 μm 90 degree	55.5 ± 1.0		82.8 ± 13.7						

All 13 included studies were in vitro investigations published up to 2025 [30–42]. The types of prostheses examined varied: five studies evaluated single crowns [33, 35, 37, 38, 42], while eight studies investigated fixed dental prostheses (FDPs) [30–32, 34, 36, 39–41]. In terms of restoration stage, eight studies focused on interim restorations [30, 32, 34, 35, 38–41], whereas five studies assessed definitive restorations [31, 33, 36, 37, 42].

In terms of restorative materials, two studies [31, 37] used cobalt–chromium (Co–Cr) alloy, whereas ten studies [31–36, 38–41] used resin-based materials, and only one study [42] used ceramic material, zirconia (3Y-TZP). This variation enabled comparison of how different material types respond to changes in layer thickness. All studies except Donmez et al. [40] and Yang et al. [34] assessed both marginal fit and internal fit. The studies also differed in their choice of reference models. Six studies [31–33, 35, 38, 40] used typodont teeth, while the other six [30, 34, 36, 37, 41, 42] employed polymethylmethacrylate (PMMA) resin master models, and only one study [39] used the nickel–chromium metal dies (Damcast NP; Damcast Dentalloy Corporation, Zhengzhou, China) fabricated by a milling machine.

For digitization of the prepared models, ten studies [30, 31, 33, 34, 36, 37, 40] [39, 42] utilizing laboratory scanners (Medit T500; Medit Corp) [30], (Artec Micro; Artec 3D, Luxembourg City, Luxembourg) [40, 42], (AIS Pro, AiditeTechnology, China), (Ceramill map 400; Amann Girrbach AG)[31], (Shining 3D ex pro) [33, 39], (Freedom HD; DOF, Seoul, Korea) [36], and (AutoScan-DS200 + ; Shining 3D Tech) [37],. Three studies used intraoral scanners, specifically the (Medit i500 v. 1.2.1, Medit, Seoul, Korea) [32, 35] and PrimeScan intraoral scanner (Dentsply Sirona, USA) [38]. Additionally, three studies [32, 35, 36] reported calibrating their scanners to ensure measurement accuracy.

Various 3D-printing technologies were represented. Three studies [30, 34, 41] employed stereolithography (SLA) printers (ZENITH U; Dentis) [30, 34, 41], while Daou et al., [31] used a selective laser melting (SLM) machine (SLM 100; ReaLizer GmbH) [31]. Many studies [32, 33, 35–37, 39, 40, 42] utilized digital light processing (DLP) printers, with specific models (MoonRay S100, SprintRay Inc, Los Angeles, CA, USA)[32, 35]; (Digident plus) [33]; (D2-120, Hephzibah, Incheon, Korea)[36], (Max UV; Asiga, Sydney, Australia), (ADT-3D-ZP-Printer-Pro-192–50, Shenzhen Adventure Tech Co. Ltd., China) [42], (Asiga UV Max; Asiga, Sydney, Australia) [39], ( SprintRay Pro95, CA, USA) [38], and (VIDA; EnvisionTEC GmbH) [37, 40].

All studies measured both marginal and internal fit, except Donmez et al. [40] and Yang et al. [34] measured marginal precision only. Three studies [30, 34, 36] specifically measured the absolute marginal discrepancy (AMD), the sum of the marginal gap and internal fit discrepancies. Layer thickness varied from 20 to 100 μm, and build orientations ranged from 0° to 90°. Additionally, measurement techniques varied across the studies. Most studies used visual inspection with microscopes for direct measurement of marginal gaps [31, 33, 37], while others employed scanning with microcomputed tomography (µCT) [30, 34, 36]. And others employed 3D superimposition techniques using digital models [32, 35], scanned with intraoral scanners [32, 35]. A detailed summary of the included studies is presented in Table 2.

Beyond descriptive characteristics, the included studies collectively demonstrate that 3D-printing parameters directly influence the marginal and internal fit of restorations. The most influential parameter was layer thickness, where thinner layers (20–50 µm) consistently produced superior adaptation compared to thicker layers (100 µm). This was evident across resin-based and metal-based restorations, although some discrepancies arose due to cumulative errors with extremely thin layers (e.g., 25 µm) or loss of precision with thicker layers (100 µm). Build orientation also emerged as a critical factor: intermediate orientations (45°–60°) minimized stair-stepping artifacts and improved adaptation, whereas extreme orientations (0° or 90°) often increased discrepancies, particularly in curved marginal areas.

Material type modulated these effects. Resin-based systems were more sensitive to variations in orientation and layer thickness due to polymerization shrinkage, while Co–Cr and zirconia restorations showed reduced sensitivity because of their isotropic material properties. Similarly, digitization methods (laboratory vs. intraoral scanners) and printing technologies (SLA, DLP, and SLM) influenced accuracy. For example, SLA systems provided more uniform energy distribution, which mitigated orientation-related discrepancies, whereas DLP-based systems exhibited greater variability depending on build angle.

Taken together, these findings suggest that parameter selection cannot be considered in isolation: restoration type, material, scanner accuracy, and printing technology collectively determine the final fit. Clinically, optimizing these variables—particularly selecting a 50 µm layer thickness and intermediate build orientation—appears critical to achieving marginal and internal gaps within the clinically acceptable threshold of ≤ 120 µm.

This systematic review critically evaluated the effect of printing layer thickness on the marginal and internal fit of additively manufactured fixed dental prostheses (AM-FDPs). Our findings consistently demonstrate that layer thickness and build orientation significantly influence prosthesis fit, with thinner layers (20–50 µm) and intermediate build angles (45°–60°) achieving optimal marginal and internal adaptation. These results align with the growing body of evidence emphasizing the importance of precise 3D-printing parameters in enhancing the clinical viability of AM dental restorations.

### Quality assessment results

Among the eight included studies, one study [35] was rated as having a low risk of bias (score = 11). The remaining seven studies [30–37] demonstrated a moderate risk of bias, with scores ranging from 7 to 9. None of the studies were classified as having a high risk of bias (scores 0–6). Common methodological shortcomings identified across studies included the absence of sequence generation, allocation concealment, and blinding procedures, as well as insufficient reporting on randomization. Additionally, several studies lacked details regarding sample size calculations and protocol registration. A summary of the individual study scores for each CONSORT item is presented in Table 4.

**Table 4:** Quality assessment of included studies

Item/author		[30]	[31]	[32]	[33]	[34]	[35]	[36]	[37]	[38]	[39]	[40]	[41]	[42]	
1	Abstract	1	1	1	1	1	1	1	1	1	1	1	1	1	
2a	Background	1	1	1	1	1	1	1	1	1	1	1	1	1	
2b	Objectives	1	1	1	1	1	1	1	1	1	1	1	1	1	
3	Intervention	1	1	1	1	1	1	1	1	1	1	1	1	1	
4	Outcomes	1	1	1	1	1	1	1	1	1	1	1	1	1	
5	Sample size	1	0	0	0	0	0	0	1	0	0	1	1	1	
6	Sequence generation	0	0	0	0	0	1	0	0	0	0	0	0	0	
7	Allocation mechanism	0	0	0	0	0	0	0	0	0	0	0	0	0	
8	Randomization	0	0	0	0	0	1	0	0	0	0	0	0	0	
9	Blinding	0	0	0	0	0	1	0	0	0	0	0	0	0	
10	Statistical methods	0	1	1	1	1	1	1	1	1	1	1	1	1	
11	Outcomes and estimation	1	1	0	0	1	0	1	1	0	0	0	0	1	
12	Limitations	0	1	1	1	0	1	1	1	1	1	1	1	0	
13	Funding	1	0	0	1	1	1	1	0	1	0	1	1	1	
14	Protocol	1	0	0	0	0	0	0	0	0	0	0	0	0	
**Risk of bias score**		(9) Moderate risk	(9) Moderate risk	(7) Moderate risk	(8) Moderate risk	(8) Moderate risk	(11) Low risk	(9) Moderate risk	(9) Moderate risk	(8) Moderate risk	(7) Moderate risk	(9) Moderate risk	(9) Moderate risk	(9) Moderate risk	

## Discussion

The findings of this systematic review highlight the complex relationship between printing parameters and prosthesis accuracy. Rather than focusing solely on numerical discrepancies, it is important to interpret these results in light of clinical acceptability and practical application. The evidence indicates that optimized 3D-printing parameters can produce fixed dental prostheses within the clinically acceptable threshold for marginal and internal fit, but several variables, such as material type, printing technology, and build orientation, modulate these outcomes.

### Impact of layer thickness and build orientation on marginal fit

The internal fit of 3D-printed prostheses is similarly influenced by layer thickness and build orientation, with thinner layers and intermediate angles generally providing better adaptation. Jang et al. [30] demonstrated that 50 µm layers at 45° achieved superior internal fit across cervical, axial, lateral, and occlusal regions (cervical: 85.3 µm; axial: 89.1 µm; lateral: 77.1 µm; occlusal: 125.7 µm) compared to 100 µm layers (cervical: 90.7 µm; axial: 102.4 µm; lateral: 100.3 µm; occlusal: 156.7 µm). Çakmak et al. [32] corroborated this trend, reporting improved intaglio trueness (lower RMS) for 50 µm layers versus 100 µm layers. Occlusal discrepancies remain a persistent challenge, particularly with thicker layers: Hasanzade et al. [33] reported large occlusal gaps for both 50 µm and 100 µm groups (420.4 µm and 697.2 µm, respectively). In contrast, Daou et al. [39] found that 25 µm layers (axial: 66.2 µm; occlusal: 137.5 µm) outperformed 100 µm layers (axial: 87 µm; occlusal: 154.5 µm), but that study’s results should be interpreted with caution, because it evaluated SLM-fabricated cobalt–chromium frameworks rather than resin-based AM systems; differences in material behavior and manufacturing physics mean those findings may not be directly generalizable to resin DLP/SLA workflows commonly used for interim and definitive restorations.

Build orientation also explains conflicting findings across studies. For instance, Park et al. [36] reported larger AMD values (169.8 µm for 50 µm layers) compared to Jang et al. [30] (71.8 µm for 50 µm layers), despite similar layer thicknesses. This difference arises from Park et al. [36].’s 60° orientation, which may increase the staircase effect in curved marginal regions. Conversely, Yang et al.[34] found no significant differences in AMD and MG between 50 µm (81 µm, 74 µm) and 100 µm layers (90 µm, 86 µm), respectively, when using SLA technology at 45°, suggesting that uniform energy distribution in SLA printers mitigates orientation-related artifacts. Another justification the resin used in yang study was UDMA base resin which offers better marginal discrepancy than PMMA used in the other studies as it generates less heat and polymerization shrinkage [43].

These findings underscore that layer thickness and build orientation are interdependent. Khaleli et al. [37] further highlighted this relationship, reporting minimal differences in marginal fit between 25 µm (59 µm) and 50 µm (58 µm) layers for metal frameworks fabricated via direct metal laser melting. The isotropic properties of metal powders may reduce sensitivity to orientation, unlike resin-based systems where angulation significantly impacts accuracy.

### Impact of layer thickness and build orientation on internal fit

The internal fit of 3D-printed prostheses is similarly influenced by layer thickness and orientation, with thinner layers and intermediate angles providing better adaptation. Jang et al. [30] demonstrated that 50 µm layers at 45° achieved superior internal fit across all regions (cervical: 85.3 µm, axial: 89.1 µm, lateral: 77.1 µm, occlusal: 125.7 µm) compared to 100 µm layers (cervical: 90.7 µm, axial: 102.4 µm, lateral: 100.3 µm, occlusal: 156.7 µm). This trend was corroborated by Çakmak et al. [32], who reported better trueness in 50 µm layers (intaglio RMS: 15.1 µm) compared to 100 µm layers (intaglio RMS: 22.6 µm) for 3-unit interim FPDs.

Occlusal discrepancies remain a persistent challenge, particularly with thicker layers. Hasanzade et al. [33] reported occlusal discrepancies of 420.4 µm for 50 µm layers and 697.2 µm for 100 µm layers. In contrast, Daou et al. [31] found that 25 µm layers (axial: 66.2 µm, occlusal: 137.5 µm) outperformed 100 µm layers (axial: 87 µm, occlusal: 154.5 µm), but the study’s.

Intermediate angles (45°–60°) appear critical for balancing precision and efficiency. Çakmak et al. [35] demonstrated that 50 µm layers at optimized angles provided the best intaglio RMS (45.4 µm) and occlusal RMS (34.7 µm) for interim crowns, outperforming both 20 µm and 100 µm layers. Steeper angles (e.g., 60°) may reduce printing time but risk geometric distortions, whereas shallower angles (e.g., 30°) could increase stair-stepping in curved regions. These findings align with Alharbi et al. [18], who noted that intermediate orientations (45° and 60°) improve dimensional stability and reduce errors associated with extreme orientations.

### Limitations and clinical implications

This systematic review has several limitations. Considerable heterogeneity was observed among the included studies regarding restorative materials, prosthesis types, printing technologies, layer thickness protocols, and measurement techniques, which make direct comparisons challenging. Most studies were in vitro and lacked methodological rigor in terms of randomization, blinding, and sample size justification, which may limit the strength of the evidence. Additionally, the absence of standardized reference models and fit evaluation methods further restricts the generalizability of the findings.

Despite these limitations, the results highlight important clinical implications. Optimizing 3D-printing parameters, particularly employing thinner layer thicknesses (20–50 µm) and intermediate build orientations (45°–60°), consistently improved marginal and internal adaptation, producing gaps within the clinically acceptable threshold of ≤ 120 µm. These findings suggest that with appropriate parameter selection, 3D-printed interim and definitive fixed dental prostheses can achieve adaptation comparable to the conventional techniques. However, validation through well-designed in vivo studies and long-term clinical trials is necessary before definitive clinical recommendations can be established.

## Conclusions

Within the limitations of the included in vitro studies, this systematic review demonstrates that 3D-printing parameters, particularly layer thickness and build orientation, have a significant impact on the marginal and internal fit of fixed dental prostheses. Thinner layers (20–50 µm) and intermediate build orientations (45°–60°) consistently yielded the most favorable adaptation, with gaps falling within the clinically acceptable threshold of ≤ 120 µm. These findings underscore the importance of parameter optimization to enhance the clinical viability of additively manufactured dental restorations. However, further well-designed in vivo studies are required to validate these results and confirm their long-term applicability in clinical practice.

## Data Availability

The datasets used and analyzed during the current study are available on reasonable requests.

## Abbreviations

**CAD/CAM**: Computer-aided design/computer-aided manufacturing.
**AM**: Additive Manufacturing
**SM**: Subtractive manufacturing.
**DLP**: Digital Light Processing
**SLA**: Stereolithography
**PMMA**: Polymethyl Methacrylate
**AM-FDPs**: Additive Manufacturing**-**Fixed Dental Prosthesis
**PRISMA**: Preferred Reporting Items for Systematic Reviews and Meta-Analyses
**FDPs**: Fixed Dental Prosthesis
**PICO**: Patient or Population, Intervention, Control or Comparison, Outcome approach
**PROSPERO**: International Prospective Database of Systematic Reviews
**MG**: Marginal gap
**IG**: Internal gap
**AMD**: Absolute marginal discrepancy
**µCT**: Microcomputed tomography.
**SLM**: Selective laser Melting
**RMS**: Root Means Square
**UDMA**: Urethane Di-methacrylate
